# How do Mental Health Service Users and Staff Think the Cost-of-living Crisis Has Impacted Mental Health in England? A Qualitative Analysis

**DOI:** 10.1007/s10597-026-01625-6

**Published:** 2026-04-25

**Authors:** Donna Clutterbuck, Monica Sood, Nisreen A. Alwan, Dianna Smith, Thomas Richardson

**Affiliations:** 1 School of Primary Care, Population Sciences and Medical Education, Faculty of Medicine, University of Southampton, Southampton, UK; 2 School of Psychology, Highfield Campus, University of Southampton, University Road, Hampshire, Southampton, SO17 1BJ UK; 3 University Hospital Southampton NHS Foundation Trust, Southampton, UK; 4 NIHR Applied Research Collaboration Wessex, Southampton, UK; 5 School of Geography and Environmental Sciences, University of Southampton, Southampton, UK

**Keywords:** Cost-of-living crisis, Inflation, Poverty, Energy poverty, Mental health, NHS, Mental health services

## Abstract

Economic variables such as debt can impact mental health. This study explored the impact of the cost-of-living crisis on mental health services users and staff, and aimed to identify potential support. Mental health service users and mental health service staff took part in interviews exploring the relationship between the cost of living and mental health, which were analysed thematically. Findings suggest that finances and mental health can be interlinked during the cost-of-living crisis, with sub-themes of ‘Economic hardship has an impact on everyone’ and ‘Hopelessness, frustration and desperate situations.’ A second theme identified was ‘Barriers to obtaining financial and mental health support’ with sub-themes of ‘Service and support accessibility,’ ‘Receiving inadequate support’ and ‘Increasing demand on services and staff pressures.’ A final theme was identified of ‘Learning from and developing on what works’. The cost-of-living crisis has had a wide-ranging impact on those using mental health services.

**Supplementary Information:**

The online version contains supplementary material available at https://doi.org/10.1007/s10597-026-01625-6.

## Introduction

Research has demonstrated that a wide range of economic variables can impact mental health at both an individual and population level. An analysis of more than 109,000 adults in Finland found an increased risk of psychiatric disorders in those of lower socioeconomic status (Kivimäki et al., 2020). A recent analysis of depression symptoms of nearly 14,000 adults in Denmark found that the prevalence of depression was four-fold for those with no education, eight-fold for those not in employment, and ten-fold for those under financial strain (Packness et al., 2025). A systematic review concluded that economic recessions are linked to a greater prevalence at a population level of Common Mental Disorders (CMDs), such as depression and anxiety, as well as elevated rates of self-harm and substance abuse difficulties (Frasquilho et al., 2015). Unsecured personal debt, such as credit card debt is also linked to a more than three-fold risk of mental health problems (Richardson et al., 2013). Quantitative longitudinal research has found a deteriorating impact of difficulties paying bills (Richardson et al., 2015) and energy poverty specifically (Bentley et al., 2023), on mental health over time.

Much research at present examining the link between financial difficulties and mental health problems is quantitative. However, some qualitative work in this area exists. In a study the UK, Barnes et al. (2016) interviewed those who attended hospital following self-harm in the context of financial difficulties, identifying overlapping difficulties around employment, debt, benefits and housing. The researchers also reported feelings around financial problems feeling unresolvable and difficulties accessing support (Barnes et al., 2016). An additional study in the UK focusing on self-harm in the context of a recession, found that finding support services could be confusing and hard to access, there was a perceived need to be self-reliant and not have to rely on others, and identified a need of practical support around benefits and debt (Barnes et al., 2017). Interviews with those experiencing financial hardship as a result of the COVID-19 pandemic in the UK found that the social isolation and loneliness of poverty impacted on mental health (May et al., 2023). Research in the UK with those with mental health problems and stakeholders has examined the impact of austerity on socioeconomic inequalities in mental health (Mattheys et al., 2018). Researchers in the USA interviewed those living in poverty with serious mental illnesses who were experiencing difficulties with debt and suggested that financial support services are often not appropriate for this population (Harper et al., 2018). This current study is situated within this context that requires further exploration.

From 2021 onwards, many countries experienced high levels of inflation in what has become known as the ‘cost-of-living crisis’ (CoL). The aim of this study was to use a qualitative approach to explore the impact of the cost-of-living crisis on the mental health of mental health service users and staff working within an area of England, to identify particular challenges which have arisen, and beliefs about possible interventions.

## Methods

### Design

This study used a qualitative approach interviewing both mental health service users and staff. It is reported in line with the Standards for Reporting Qualitative Research (SRQR) guidelines.

### Participants

Study eligibility was based on being either an adult reporting experiencing mental health problems, or a staff member working within the National Health Service (NHS) or voluntary sector,. Additionally, participants needed to be aged 18+, and able to provide informed consent. Inclusion criteria also required being located in the counties of Hampshire, Dorset or the Isle of Wight on the south coast of England (due to the study being funded to research the health needs of this specific area). Study advertisements were distributed through staff and service user channels by participating NHS trusts and voluntary organisations including food banks. Potential participants were encouraged to follow a link to the participant information sheet (PIS) or to contact the research team directly for more information and check eligibility. Those who completed the online consent form and confirmed eligibility were invited to take part in a semi-structured interview. The characteristics of the service user sample are shown in Table 1. There were a range of diagnoses including common mental health disorders, such as depression and anxiety disorders and Post-Traumatic Stress Disorder, and more serious mental illness include bipolar disorder, personality disorder, and schizoaffective disorder. Table 2 shows the characteristics of the staff sample.

**Table 1:** Service user characteristics

	Number service users (%)
Gender	
Female	5 (45)
Male	6 (54)
Age	
20–29	1 (9)
30–39	2 (18)
40–49	2 (18)
50–59	3 (27)
60–69	2 (18)
70+	1 (9)
Ethnicity	
White British/White/White other	11 (100)
Currently using mental health services?	
Yes	9 (81)
No	2 (18)
Diagnosed mental health condition?	
Yes	9 (81)
No	2 (18)

**Table 2:** Staff characteristics

Gender	
Female	9 (100)
Male	0 (0)
Age	
20–29	4 (44)
30–39	2 (22)
40–49	2 (22)
50–59	1 (11)
60+	0 (0)
Ethnicity	
White British/White/White other	8 (88)
British Asian/Asian	1 (11)
Currently working in mental health services?	
Yes	6 (66)
No	3 (33)

### Interviews

Interviews were completed with eleven mental health service users and nine service staff. Semi-structured interviews were employed as these ensured that key questions were asked, while also allowing room for flexibility depending on participant answers. The interview topic guides (see supplementary material) were developed by the research team with patient and public involvement (PPI) representative input. The service user topic guide explored financial difficulties, mental health and the use of financial and mental health support and services. Staff were asked questions surrounding the mental health and financial difficulties of their service-users, service demands and ways of improving services. Demographic information was collected from all participants at the beginning of the interviews before recording commenced.

Interviews took place between April and June 2024 via MS Teams video or phone call. The telephone option was offered to ensure that interviews were inclusive and responsive to the needs of participants. All interviews were audio and/or video recorded and transcribed using the features embedded within MS Teams. Transcripts were then checked for accuracy, corrected and anonymised prior to analysis. Interview participants were offered a £20 shopping voucher as a thank you gesture for taking part.

### Analysis

Thematic analysis was employed to analyse interview transcripts, guided by the approach outlined by Braun and Clarke ( 2021). Initial inductive coding was completed by DC, aided by NVivo. MS completed additional, independent coding on a sub-sample of transcripts to ensure intercoder reliability. DC and MS were broadly in agreement in relation to codes. Any discrepancies were minor and were resolved through discussion and coming to a consensus. Once coding had been completed, DC then began grouping together of similar and related codes in order to form themes. In some cases, some of these themes were seen as related, but still distinct, and so formed sub-themes relating to broad themes. DC then started to build a draft theoretical framework, looking at the themes identified in the staff and service users interviews separately. It was clear that there were some commonalities between service users and staff members in terms of broad themes, but there were also some distinctions between the participant groups at the sub-theme level. DC then consulted with the team to check agreement of themes before starting to write-up. The refinement of themes and sub-themes was an iterative process that continued with input from all authors while drafting this paper. Authors on this paper are from diverse research disciplines. Bringing together the expertise of all authors enhanced the reflexive analysis process.

### Ethics

Most participants were already in contact with NHS mental health services and were advised to contact if needed. The study participant information sheet and debriefing form include details of services for both mental health and financial support. Ethical approval for this study was obtained from NHS research ethics committee (23/SC/0413) and the University of Southampton ethics committee (84818). If a participant seemed upset during an interview, the researcher would ask them if they were OK to continue before proceeding. No participants expressed wanting to end an interview early due to distress. The researcher would then check in again at the end of the interview. Regular team meetings were set up, and the project Principal Investigator, who is a clinical psychologist, was available after interviews to debrief about any concerns about participants.

## Results

**Fig. 1:**
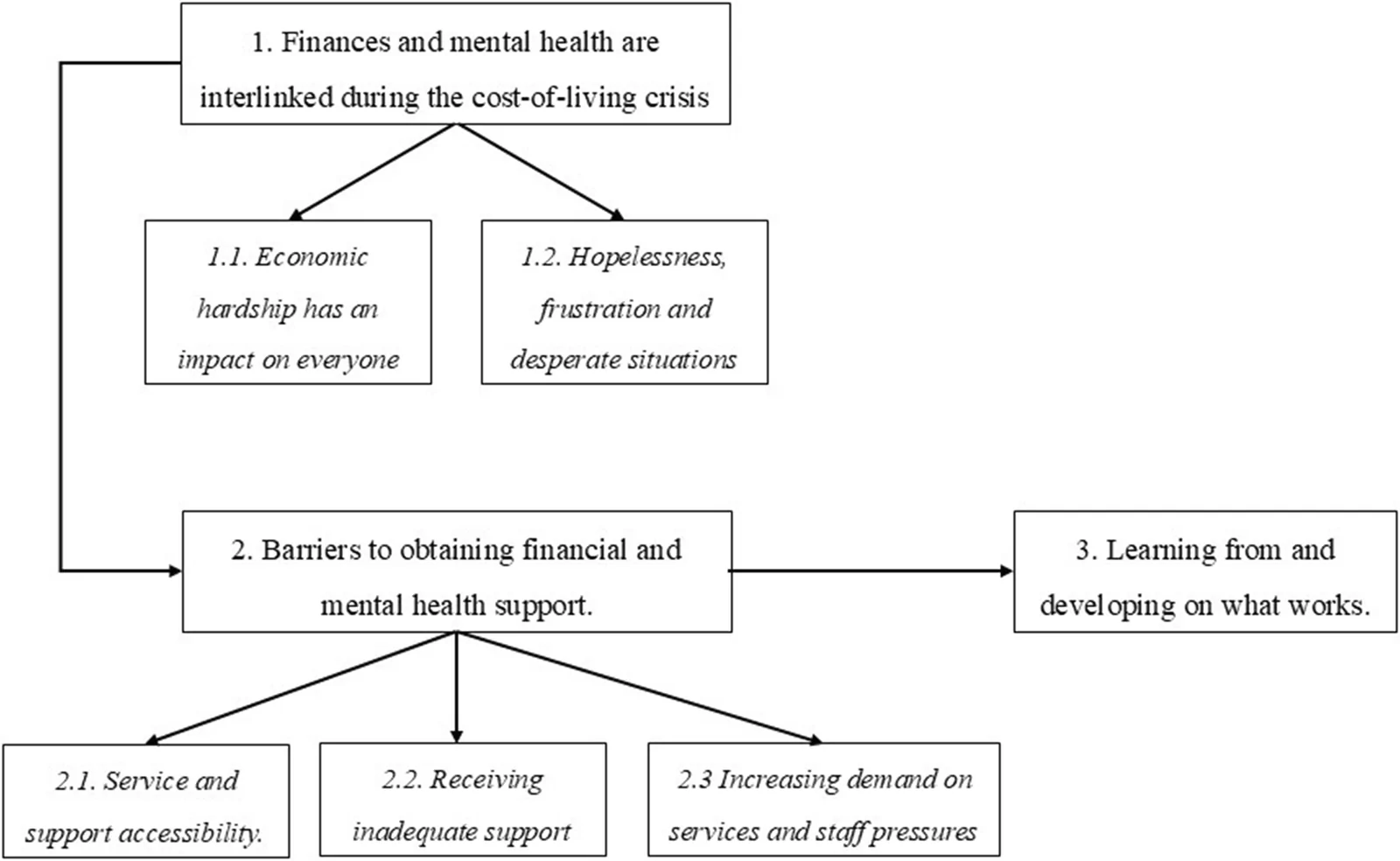
A thematic map of the inks between the themes

The following sections focus on the three broad themes identified during analysis from interviews with both staff and service users: (1) Finances and mental health are interlinked during the Cost of Living (CoL) crisis, (2) Barriers to obtaining financial and mental health support, (3) Learning and developing on what works These themes provide a picture of how the CoL crisis is impacting on the mental health of people living in Hampshire, the Isle of Wight and Dorset, and the services that aim to provide support. It should be noted at the outset that some service user interviewees were audibly upset during the interviews, and the protocol as described in the ethics section above was followed. Some participants shared that they had not felt listened to previously, or managed to express the breadth of their experiences to others. These comments alone revealed the hardship and desperation experienced by some. Illustrative quotes are shown in Table 3, with a thematic map displayed in Fig. 1.

**Table 3:** Additional illustrative quotes

*Theme*	*Quote Number*	*Additional Illustrative Quotes*
1. Finances and mental health are interlinked during the CoL crisis. Sub-theme: 1.1. Economic hardship has an impact on everyone.	A	I’ll definitely, um, eat more rubbish. I don’t eat. I do go to the food bank more and I try and eat some healthy stuff, but generally healthy stuff is more expensive in my opinion… I do little shops anyway because I’ll never have a lot of money at once. I make an effort to eat like a couple of good meals a day or some fruit weeks or something. But yeah, definitely I’m more likely just to pick up something cheap and cheerful and easy… (110, service user, 50–59, female).
	B	Food is more basic, more just what we need is. It’s not so many treats and stuff like that for the kids so much anymore. (104, service user, 30–39, male)
1. Finances and mental health are interlinked during the CoL crisis. Sub-theme 1.2. Hopelessness, frustration and desperate situations.	C	…my feeling now is I just like currently managing day-to-day. So any debts or anything else like that…just get well, not even really thought about to be perfectly honest…I know
		I’d feel better if I went through and sorted it all out. I just can’t be arsed. I’ve just not got the motivation…to do that. (110 service user, 50–59, female)
	D	I think for me it’ll be further down the line that the crisis will come because I won’t have the savings that I need in my older years because I’ll have spent them if this condition continues
		and I lose my job or I lose my lodger or whatever,…. That concerns me…cause I don’t have very good pensions. (102, service user, 50–59, female)
	E	Spend loads of money. Feel guilty about it. They then don’t want to use the thing they spent the money on they feel guilty about spending the money and again it feeds back into the cost-of-living
		crisis… I think it can make those kind of impulsive purchasing decisions more likely. But…because of the cost-of-living crisis, people… feel more guilty about them… So then the money is spent
		and they’re not even getting the kind of positive psychological benefits of doing something they enjoy. (115, staff, 20–29, female)
	F	… we are seeing that people are mentioning their mental health more and more in the referrals as well. And I know…we don’t deal with this, but… we have to make the GP aware about this.
		…we have people who come and sometimes have a offload on us. You know how this has changed things for them. And then it’s…getting… them to the right place. So in that way, yes, we
		have seen that difference into our service. (105, staff, 20–29, female)
	G	So this year I’ve…been hospitalised twice from overdoses of medication….It was not suicidal attempts, but suicidal thoughts that were getting a bit more physical and real. … it’s definitely been a contributor in that respect because I think it’s… just part of a number of things that can exacerbate existing conditions (120, service user, 40–49, male).
	H	… because we’ve had to tighten our belt quite a lot and we haven’t been able to do some of the things that maybe keep us happy in that. You know, we don’t really go out for dinner as much. We don’t go in and do all the things that were the things that sort of kept us going, I suppose… it feels a little bit like it’s just a bit of a drag rather than like…let’s go and do this, let’s go and do that. (126, service user, 50–59, female)
	I	… often…if we’re doing treatments or CBT, we’re maybe encouraging people to kind of go out and…do things for their mental health and …healthy lifestyles…food and…there comes that element of I can’t afford to eat healthily or I can’t afford to go to the gym or…do things nice things that I want to be able to do. And it’s kind of put in a…lot more barriers in place to help people get back to …to feeling well. (111, staff, 30–39, female)
	J	… sometimes…my colleagues will bring cases to me, labelling it as health anxiety or GAD, when actually it kind of boils down to the cost-of-living crisis, where it’s not GAD in the sense that they’re kind of worrying loads about lots of different things. They’re having a lot of worries, but they’re about finances. They’re about how am I going to pay my bills and…I don’t necessarily think that’s a mental health problem… of course you’re stressed because you’re worrying about how you’re gonna heat your home and you know, kind of feed your children (115, staff, 20–29, female).
2. Barriers to obtaining financial and mental health support: Sub-theme:2.1. Service and support accessibility.	K	Even the Universal Credit… you can tell them that you’re not well. You can give them sick notes and then they send you a form that you fill out…but I don’t see how someone can just look at a form and judge that. If your doctor said that you can’t…I don’t understand. (104, service user, 30–39, male)
		L If you’re already struggling…you might be more focused on making ends meet before thinking about mental health support and also people who might be working multiple jobs might not have the time and that is also common… …. So I think more deprived areas will be facing these…barriers to access …because they haven’t got time and also if the priority is something like housing, they might not think about the mental health support. And back to that Maslow hierarchy. (119, staff, 20–29, female)
2. Barriers to obtaining financial and mental health support: Sub-theme: 2.2.Receiving	M	… I think some GPs have got to do more to help people…. There was a time when I was really low and I …thought like I’ve had enough, you know. But for a glimmer I thought I’m going to phone the GP and speak to someone. And they just said to me We can’t deal with this. You need to phone 111…And I don’t think that’s great. (104, service user, 30–39, male)
inadequate support.		
2. Barriers to obtaining financial and mental health support: Sub-theme: 2.3. Increasing	N	… among myself and my colleagues, especially over Christmas, I think everyone gets quite burnt out and you do reach a point where I have supervisees saying …I don’t know what to say to them because CBT isn’t gonna help them with this… challenging your thoughts or you know kind of changing your behaviour isn’t going to change the fact that you can’t change your bills or telling people to do things like worry management…just problem solve it or use worry time…It feels a bit insincere…this is a genuine problem that is affecting your day to day life… It’s not really a mental health problem. It’s affecting your mental health, but the root cause of it is completely outside of their control, completely outside of our control as well. (115, staff, 20–29, female)
demand on services and staff pressures.		
What helps andwhat is needed 3. Learning from and developing on what works	O	A little bit more…support and not…just in money… Even if someone gets back to you quick enough. At least you feel like you’re being listened to…or someone’s heard you. So I don’t think money’s the…answer to everything…it wouldn’t have made my mental health any better. It would have just maybe eased a little bit pressure for us for six months maybe…. … if someone just gets back to you and then you…sort of feel like you’re slightly important, still a human being, then I think…that would be good,. (104, service user, 30–39, male)

### Finances and Mental Health are Interlinked During the CoL Crisis

#### Economic Hardship Has an Impact on Everyone

Financial hardship, in varying degrees, was prominent in the narratives of both service users and staff. Although all service user participants were impacted by the CoL crisis to some degree, it was clear that some were impacted more than others.

*I mean*,* compared to a lot of people you know our problems are…quite small. But yeah*,* I mean*,* we’ve definitely had some issues during the cost of…living crisis.(126*,* service user*,* 50–55*,* female)*.

Some participants talked about being able to ‘adapt’, ‘budget’ or live within their ‘means’. Sacrifices for these participants often came in the form of cutting back on leisure activities. For participants sat at the other end of the continuum, hardship was much more acute. Some participants adapted how they shopped for food This resulted in making unhealthy food choices, doing smaller shops, forgoing ‘treat’ foods and choosing affordable options (see Table 3, quotes A and B). Some participants, however, talked about their use of food banks and pantries. Some also relied on vouchers to fuel their homes.

*Always have to go to the food bank. Not just once a month but nearly every week. (122*,* service user*,* 60–69*,* male)*

Others talked about reducing clothes shopping and shopping second hand where possible.

*All the clothes we get for [daughter] and stuff are all off*,* like Vinted and stuff like that. We use that a lot rather than shops. (106*,* service user*,* 30–39*,* male).*

A few participants mentioned the expense of running a car and the impact this had on getting to work, taking children to school and seeing family. Financial struggles sometimes resulted in essential bills being paid late and rent arrears, and one participant admitted that they resorted to shoplifting.

*And on occasions…I’ve had to shoplift.* (122, service user, 60–69, male)

Additionally, social activities were cut by the majority of participants. Financial difficulties within this sample were sometimes compounded by losses or changes to salary and ill-health.

Staff also noted that service users are requesting more financial help from services, including referrals to food banks and benefit application support and advice. This subtheme has detailed how economic hardship in the CoL was experienced by all service users, but was felt on a continuum. Some were able to manage through adjustments to budgeting and making leisure activity sacrifices. Others were compelled to make cut back to essentials.

#### Hopelessness, Frustration and Desperate Situations

Financial difficulties created a sense of hopelessness and frustration that had serious impacts for some. Most service user participants felt that financial difficulties had an impact on their mental health or wellbeing in some way and for some, this impact was much more acute. Some service users described the impact on their mood and sleep which worsened at critical times.

*[Financial difficulties] Made me feel very low (106, service user, 30–39, male)*.

*Certain days where bills are meant to be due. I find I don’t sleep very well. (104*,* service user*,* 30–39*,* male)*

Feelings of being trapped, isolated or lonely were apparent from participant interviews, as were feelings of hopelessness and being unmotivated.

*Ohh can you imagine*,* my age? I should be retired. I mean*,* more than ever…I don’t know how to get out of some of it. (116*,* service user*,* 60–69*,* male).* Service users talked about stress, worry and anxiety surrounding finances. This included worries about the future (Table 3, Quote D).

Some suggested that this resulted in being unable to focus or concentrate on anything else. Additionally, one service user participant suggested that his partner’s anxiety around finances impacted him and caused frustration.

*… sort of the more knock-on effect that it has on my partner and how she feels about it that has a bit more of an impact on me. (120*,* service user*,* 40–49*,* male)*

Guilt around spending or using household appliances was also cited by a few participants.

,,, *sometimes I feel…guilty because I’m at home a lot now because I can’t really get out and about anymore.…so I sometimes I’ll just sit it in in*,* in silence. So I won’t turn the TV on. (104*,* service user*,* 30–39*,* male)*

Staff participants echoed much of what service users described in terms of mental health impacts. Some stated that almost all service users mentioned the impact of the CoL crisis on their mental health. There was acknowledgement of a general increase in stress and anxiety among service users. Staff noted that although worry and low mood have always been linked to finances amongst service users, this increased during this period of financial crisis.

*I think stress is probably the number one*,* but I think sort of feeling quite hopeless as well…there’s not a lot in their control to be able to sort of manage that side of things because that’s how it is. So feeling quite helpless and hopeless about the situation and not sort of having any answers or any solutions. (113*,* staff*,* 20–29*,* female)*

There was acknowledgement that sometimes people feel guilty for spending money on themselves, and that feelings of hopelessness and frustration associated with financial insecurity or uncertainty are likely to be impacting mental health too (Table 3 quote E).

Staff from some non-mental health organisations suggested there had been an increase in requests for mental health support during this time. This includes staff from food aid, general advice services who see members of the public when they need help with basic needs or benefits and debt advice. During meetings with service users, the challenges faced by these clients sometimes included mental health spontaneously being mentioned (Table 3 quote F).

Two service user participants had been hospitalised for mental health within the few months leading up to the interview. Both saw financial difficulties as contributory factors to these hospitalisations.

*…I’ve recently had a…seven week stint*,* at a psychiatric ward just cause lots of things built up. Obviously.…Money was…part of things that caused the relapse…the high pressure to maintain my work. If I don’t*,* then…we can’t pay our mortgage and all that stuff. So there’s a lot of pressure (106*,* service user*,* 30–39*,* male)*.

As more severe outcomes of poor mental health, self-harm and suicide were less often discussed compared to general anxiety, stress, or depression. Suicide or self-harm were mentioned within four service user interviews. Sometimes this was talked about as something that was understandable under pressurised conditions.

*A few sessions ago…with [therapist] Umm*,* I said to her*,* I understand why some people consider suicide because it’s just something*,* I understand all the pressures (116*,* service user*,* 60+*,* male)*.

But other times self-harm or suicidal thoughts were talked about as something that had occurred, resulting, at least in part, from financial difficulties.

*Basically I self harm*,* I do a lot of self harming. (122*,* service user*,* 60–69*,* male)*

This was also the case for some people with existing mental health diagnoses (Table 3 Quote G).

One staff participant described how financial difficulties could exacerbate suicidal feelings in some people.

*And not knowing if you’ve got enough money to get through the next week*,* um obviously has a detrimental effect on people… that are feeling suicidal. …You know I can’t afford to live. So what’s the point? And yeah*,* I definitely think it’s is quite apparent that it’s…having a high*,* high impact. (107*,* staff*,* 30–39*,* female)*

Restrictions on spending meant that some service users were unable to do things to create happiness because of affordability. Participants talked about not being able to afford to spend money on themselves or having to concentrate on being able to afford mundane things (Table 3, quote H). Similarly, staff participants felt that financial difficulties impeded on the ability of some to improve their wellbeing (Table 3, quote I).

For some service users, financial difficulties occurred within the context of other life circumstances, changes and experiences, such as bereavement or changes in medication. This meant there was some difficulty identifying the main cause of poor mental health. For others, mental health problems were seen as being exacerbated by poor physical health, particularly if ill-health resulted in being unable to work.

*And I think that’s where the mental health started from the pain*,* from the pain I was always in and not being able to do things (104*,* service user*,* 30–39*,* male).*

Some staff members suggested that stress surrounding finances were sometimes labelled as mental health problems when, in fact, this stress can be seen as a ‘normal’ response to economic difficulty (Table 3, quote J).

The CoL and financial difficulties fostered a sense of hopelessness and frustration. This resulted in stress and stress and anxiety, and less often, self-harm and suicidality. Economics also prevented engaging in behaviours that facilitate improvements to wellbeing.

### Barriers to Obtaining Financial and Mental Health Support

#### Service and Support Accessibility

The accessibility of both mental health and financial support services was a barrier for some, with overlaps between accessibility for both finances and mental health, hence why these were combined into a single theme. Most service users talked about not having to pay to use NHS mental health services. However, two participants mentioned that limited funds meant that they were unable to explore whether private therapy might be of benefit to their mental health.

*I think I would really benefit from having*,* potentially… a private therapist*,* but then financially*,* it’s just not feasible and it’s like*,* what would the impact of having a private therapist have on me…would that be…really beneficial to me*,* but I just don’t know and it’s not something that I can explore at the moment (118*,* service user*,* 20–29*,* female).*

Some participants described the difficulties they faced when attempting to obtain financial support to help meet their living costs, such as housing and food. Participants experienced challenges when attempting to get welfare benefits, such as Universal Credit (UC) and Personal Independence Payments (PIP) to ease financial difficulties(Table 3 Quote K). Some felt that the benefits that they did receive were insufficient. Others talked about unsuccessful benefit claims or not meeting the entitlement criteria, for example with Employment and Support Allowance and PIP. There were also issues related to the application process for welfare benefits in terms of completing the forms, response times, as well as suggestion of unfairness embedded within the application process.

*Just having to justify yourself….They won’t just accept the fibromyalgia… You constantly have to justify it and get forms*,* work capability assessment… even …the PIP thing. I just think it was a waste of time me filling it out. (110*,* service user*,* 50–59*,* female)*

Some service user participants suggested that they had not sought mental health and/or financial support. Reasons for not seeking financial support included not knowing where to find support, feeling unentitled to help, relying on informal support from family and using online money-saving tips and budgeting tools.

A few service user participants considered “everyone” to be experiencing the same difficulties. One participant suggested that she had not reached out for mental health or further financial support as her friends were having the same problems and felt that others were worse off than her. This was despite experiencing stress as a result of her difficulties.

*No*,* I haven’t really looked into it to be honest*,* [laughs] cause I think a couple of my friends*,* they’re… stressed as well about all this cost-of-living and I think…it’s stressful for everyone to be fair. (124*,* service user*,* 40–49*,* female*.

Here, it is worth noting that some people identified ‘stress’ as a mental health concern,, while some staff members de-emphasised stress as a mental health issue, discussing it in contrast to survey-measured Generalised Anxiety Disorder (GAD).

Staff participants were also mindful of the potential inaccessibility of some services and logistical barriers that might prevent some from attending services. These included transportation costs and being unable to attend appointments. One staff member suggested that a lot of the support available was accessible online, which meant that people who were unable to access a computer or tablet were excluded. There was also suggestion that some might be more focused on their financial situation than prioritising their mental health (Table 3, Quote L). This does not recognise the potential connection between financial insecurity and poor mental health, though the awareness of competing priorities is relevant in this research.

Staff felt that there was a lack of awareness of the support available for mental health and/or financial difficulties, which some staff participants thought was particularly true for people who had not previously accessed support. This demonstrates an area for potential impact, to ensure support for both financial problems and mental health are widely advertised. The CoL did not directly impact ability to access NHS support. However, a lack of financial resources meant that exploring other sources of support – such as private therapy – became unattainable, and travel and IT costs for NHS treatment could nonetheless be problematic. Obtaining welfare benefits can be problematic and cumbersome. Additionally, downplaying of difficulties reduced acknowledgement that support may be required. These factors, combined with the lack of awareness, digital literacy issues, and the indirect costs of accessing support services created inaccessibility to obtaining help needed.

#### Receiving Inadequate Support

Inadequate support worked as a barrier to obtaining the help that was needed. Some service users described negative interactions with mental health staff including aspects of role reversal with staff also struggling. These experiences are likely to have impacted how they perceived mental health services:

*I made my counsellor cry…I ended up kind of supporting her not the other way around…but …that was not to do the with the cost of living…That was kind of to do with everything as well. (126*,* service user*,* 50–59*,* female)*

Two participants were currently receiving support from mental health crisis teams. Both felt that they were getting the support needed under the care of these teams. However, one participant felt that prior support was lacking and the other held the perception that a patient had to be considered to be ‘high need’ to get support. This latter participant was concerned about what would happen when discharged from the crisis team.

*There’s still the element of once you go into the crisis team*,* where’d you go after that? And you end up sort of being bounced around various departments. (120*,* service user*,* 40–49*,* male)*

Other service user participants talked about the difficulties experienced when trying to engage with primary care. Some talked about difficulties obtaining a General Practitioner (GP) appointment.

*Trying to get a doctor’s appointment, just like raging nightmare*. (110, service user, 50–59, female)

While some service users acknowledged that their GPs were supportive once they were able to get an appointment, another talked about not feeling supported by their GP, and being signposted to other NHS support that does not include face-to-face contact (Table 3, Quote M). Being bounced around different services, receiving insufficient support, negative interactions with professionals and poor communication between services created a barrier to obtaining support.

#### Increasing Demand on Services and Staff Pressures

A lack of NHS and mental health service resources coupled with increased need were impacting support that could be provided. As described above, participants described issues with trying to engage with primary care, others felt that support had been ‘diluted’ because of a lack of funding. Two service users, who worked within healthcare themselves, mentioned staff wages and NHS resources in their interviews. One suggested that staff morale was low, and this was impacting on their mental health.

*…and I think…maybe getting to the point where we can either get more staff or people can be more realistic of what’s achievable for each person. Because I think at the moment I just don’t see anybody around that’s happy in*,* in their work because they’re just up against it all the time and…that affects your mental health. (126*,* service user*,* 50–59*,* female)*

NHS and voluntary service participants felt increasing service demands throughout the CoL crisis. Some mental health service staff noticed an increase in referrals during this period, which increased waiting time for mental health services. Though they could not be sure that this was caused directly by the CoL crisis, some felt it was a contributory factor.

Non-mental health and voluntary service staff had also noticed an impact. One staff member from the voluntary sector, for example, talked about how service users were opting to engage with free and subsidised services as opposed to paid services that the charity was offering. This was causing financial repercussions for the service at a time when fundraising income was low and there was a shortfall in voluntary staff.

*I can sum it up with*,* we should be able to expand to cope with demand and we’re contracting because fundraising has gone down the pan. (103*,* staff*,* 50–59*,* female)*

Similarly, one NHS staff member suggested that the increase in waiting lists in the voluntary sector as well as the constraint in resources in healthcare meant a limit in the support (such as number of sessions), that could be provided. This, coupled with the reduction in those with resources to access private services, could be impacting on NHS services.

There were limits to what services could provide. Healthcare staff noted an increased amount of signposting was required. This meant that they needed to be well-informed of the support that was available. There was also a sense of staff helplessness. Staff felt that there was only so much that counselling therapies could provide for people experiencing financial difficulties. This reflects the discussion above regarding competing priorities for help-seeking, between financial or welfare support as more important compared to mental health support. There is an inherent relationship between financial worries and poor mental health, and it should not be a situation of help for only one or the other, as prevention of either poor mental health or reduction in financial pressures can benefit the other.

*Like*,* how do we work on*,* for example*,* a standardized…worrying management intervention or standardized behaviour activation intervention when the person that we’re seeing doesn’t know how they’re gonna feed their kids next week or they don’t know if they’re gonna have to move house or lose their house. (119*,* staff*,* 20–29*,* female)*

Staff sometimes felt conflicted in the ways that service users could be advised when talking about methods to improve mental wellbeing, and sometimes found the restrictions in what they could provide to service users difficult (Table 3, Quote N).

Funding, an increase in referrals and waiting times as well as demands on staff working within services was perceived as resulting in diminished support for service users.

### What Helps and What is Needed– - Learning From and Developing on What Works

Despite pressures and the level of hardship experienced, there were some encouraging avenues of support available to those in need as well as some identified ways of facilitating help.

Food banks and fuel vouchers, and the government-funded Energy Bills Support Scheme/CoL payments were invaluable to some service users.

*We have got the cost-of-living payments. So that has helped a lot… Especially like with what Council tax for example. ….* (124, *service user*,* 40–49*,* female*)

Service users also accessed and valued support and advice that came from their personal networks as well as from professionals, including clinicians, charities, voluntary organisations and faith leaders.

*…at the moment I’m sleeping on the floor on a mattress. I’ve got a charity that’s going to deliver it on Thursday…a sofa and goes into a bed. (122*,* service user*,* 60–69*,* male)*

Service users also accessed online financial advice and support techniques about finances. However, some service user participants expressed a need for more support. A few felt that this should go beyond financial support, and be personalised so that people feel listened to (Table 3, quote O).

Staff discussed ways in which they were trying to help people with financial difficulties. This included providing food bank vouchers, help with formal letters and applications, and signposting to other services for specialist support. There were also psychotherapeutic forms of support available within some services, such as group therapy, working with service users to problem-solve and, one staff member mentioned that their service had a workshop geared towards finances as well as a module similar to SilverCloud^1^ online CBT interventions.

*We also offer a financial workshop. It’s quite new….So that’s having a bit of an uptake and that’s using low intensity CBT in the context of financial difficulties similar to the SilverCloud content. (119*,* staff*,* 20–29*,* female)*

However, some staff members felt that their service could do more: finance discussions should be routine, and more education for staff around financial difficulties and support options should be available.

*I think we need to do more open and honest conversations about people’s finances and try and get families more involved if they consent to it. I think families need to be more aware of…that and I think we need to do more work around. (107*,* staff*,* 30–39*,* female)*

Dedicated services for managing money, and services that provide a centralised hub for support and multi-agency support were also considered as warranted by staff participants.

Some facilitators to support were seen as crucial by both staff and service user participants. Both expressed a need for more accessible and free services, more practical support, as well as more awareness of options available, both within services and in the community,.

*…. It does come down to just practical support because that’s what you need to get through to feel like a little bit better. But then obviously you need steps in process to then help you move on and help yourself. (110*,* service user*,* 50–59*,* female)*

Service users valued welfare benefits as well as support from professionals working in healthcare and the voluntary sector. Staff were able to provide limited practical and psychotherapeutic support. However, a need for more, free, dedicated, practical and accessible support, as well as awareness and education surrounding this support was identified.

## Discussion

This study employed qualitative interviews with staff and service users to explore how the CoL crisis has impacted mental health. Overall, the findings suggest that the CoL crisis, consisting of high inflation costs in particular for energy and food, has worsened the mental health of service users in the UK and strained services during this time. This aligns with previous quantitative research showing, for example, that recessions increase the prevalence of mental health problems (Frasquilho et al., 2015). Although there is a large body of previous research demonstrating a link between financial difficulties and mental health problems, few studies explore the specific mechanisms of how and why these are related. Existing research often focuses on a few specific variables rather than openly exploring these links using qualitative methodology as in this study. This study uniquely demonstrates via the views of service users and staff the complexity of this relationship, that there are multiple different and interacting mechanisms by which financial difficulties, exacerbated by the CoL crisis impact mental health.

Some of these key factors include reduced social support, having to cut back on costs such as holidays, cars, clothes and preventing spending on activities which would improve mental health. Previous qualitative research has found that loneliness from poverty impacted mental health during the COVID-19 pandemic (May et al., 2023), and quantitative research with those with bipolar disorder showed that having to go without essential items worsens symptoms of depression (Richardson et al., 2017). Participants reported the CoL crisis impacted sleep, increased suicidality, self-harm and hospital admissions in the context of hopelessness, guilt and desperation. This is in line with previous qualitative findings about self-harm in the context of economic difficulties noting a theme of financial problems feeling ‘unresolvable’ (Barnes et al., 2016), as well as quantiative research on the role of hopelesness in linking financial hardship and mental health (Frankham et al., 2020).

The Col also directly impacted on ability to receive support and treatment in terms of being unable to have the option to pay privately, difficulty affording travel or digital requirements for appointments. The cost-of-living crisis has increased pressure on staff within this study due to increased demand on already stretched mental health services, with staff interviewed reporting that third sector organisations having to reduce offer due to financial pressures. Data on NHS Talking Therapies shows an increase in referrals from 1.46 million in 2020-21 to 1.81 million in 2021-22, staying relatively stable since then at 1.76 million 2022-23, 1.83 million in 2023-24 and 1.81 million in 2024-25 (NHS Digital, 2025). However, this does not capture the work of secondary care mental health services, and it is unclear if they have experienced increased demand. It may be that while referral numbers have not increased, financial difficulties may have intensified the complexity of the work, during this time, leading to services feeling stretched; previous research shows that those in deprived areas need more sessions of psychological therapy to recover (Finegan et al., 2020), and it is possible that more sessions are needed during the CoL crisis.

The theme of ‘what helps’ suggested specific actions such as continuing support via fuel vouchers and, food banks and CoL for those experiencing financial hardship, the importance of involving wider community organisations such as charities and faith organisations. This also stressed the importance of integrated interventions where possible, with finances and mental health being tackled at the same time, with specialist financial support services needing to ‘go beyond’ just financial support and have this tailored of the individual and feeling heard and validated. The difficulties identified around access to support are consistent with previous qualitative research (Barnes et al., 2016). Despite treatment being provided free under the NHS, poverty still represented a barrier to attend treatment due to transportation costs and difficulty finding time to access therapy with some occupations. This suggests that flexibility around appointment times may help improve outcomes and engagement for those struggling financially. The use of reduced travel cost initiatives such as disabled railcards and bus passes (Citizens Advice, 2026), as well as increasing awareness that those on certain benefits may be able to claim back costs for appointments (NHS, 2023). Services should consider providing or liaising with charities which may provide digital devices to reduce the impact of digital poverty and low digital literacy and allow attend online appointments. Building in psychological therapy is perceived as difficult when there is significant financial hardship, and people needing to prioritise basics like feeding children over attending therapy. This reinforces the finding that outcomes from psychological therapies for depression and anxiety are worse for those who live in poorer neighbourhoods in England (Finegan et al., 2020), and highlights the need for flexibility and holistic care to support the mental health of those experiencing financial hardship.

Difficulty in accessing financial support and a need for practical advice and support around finances, has been found in line with previous research (Barnes et al., 2017). There were some examples of integrated interventions, such as an NHS-based therapeutic group focused on financial difficulties; however, generally participants described mental health and financial advice services as operating separately from one another. A key issue identified here is that simply directing to other services does not appear to be enough as service users find it hard to navigate this and feel ‘bounced’ between; different services. The concept of ‘no wrong door’ (Bell & Pollard, 2022), which has been applied to some mental health services, should also be applied to support for finances as well. This study specifically highlights this is a need for before, during and after mental health crises such as inpatient admissions, so increased awareness in services users and training for staff on the ‘mental health breathing space’ (Money and Pensions Services, 2026) which pauses interest and debt collection activities during such a crisis, is needed.

It is necessary for professionals to have open and honest conversations about money with their service users., Furthermore, health professionals identified in the ‘what helps’ theme the need to have awareness training on the link between money and mental health. This is in line with the Money and Mental Health Policy Institute which recommend that mental health practitioners routinely ask about finances, receive training, provide or refer to specialist help (Clarke, 2017), and have closer integration between money/debt advice and NHS Talking Therapies during the CoL crisis (Bond, 2023). Reluctance to seek help due to a sense that it was normal and that everybody else was struggling suggests that those struggling with finances and mental health problems may be reluctant to seek help as this situation is normalized and widespread within communities. There was also an issue of not seeking support until a crisis point. It is therefore important for clinicians to encourage and support access to financial advice. Public health campaigns on the link between financial difficulties and mental health problems may be useful to advertise available money/debt advice, encourage early help-seeking, and thereby facilitate early intervention.

This study is limited by a small, heavily female and white sample from a small geographic area in England. Likewise, data related to service user employment, housing, income, welfare benefit status or time open to mental health services was not collected. This means inferences related to these characteristics cannot be made. The interviews were conducted online or via phone rather than in person which may have impacted results as those without access to a telephone or the internet may have been excluded. However, online interviewing also offers the benefits of flexibility to fit around work and allows participants to feel comfortable in their own environment (Irani, 2018). Though it is possible there may have been a bias towards participants giving socially desirable responses in the interview, interviewing online may have reduced such as bias. It is possible that there is a recruitment bias whereby those who were struggling financially were more likely to take part in the research, especially as they were given voucher payment for participation. Not all participants were current users of NHS mental health services. The perspectives of carers were not sought which may have led to diverse findings. Due to these limitations, it is not possible to generalise these findings more widely: We can conclude that the cost-of-living crisis has had an impact on mental health services users from this part of England, but cannot conclude about impacts beyond this region and at a general population level. Future research covering a broader geographical range and diversity of participants is warranted. Given the significant but limited findings around the impact on psychological therapies specifically, further research interviewing CBT therapists and psychologists working in the NHS about how financial difficulties impacts their work with clients would help to identify adaptations which may improve access and enhance outcomes from those with mental health problems living in deprived areas.

## Conclusion

The CoL crisis has had a wide-ranging impact on those using mental health services in Wessex as well as impacting services in this area directly. This study highlights the complexity of the link between financial difficulties and mental health, with mental health service users reporting that the CoL impacted nearly every aspect of life, as well as impacting the services, organisations and staff they rely on for support. Integrated interventions, increased awareness of existing supporting schemes, training for mental health staff and closer working between mental health and money, debt and benefits advice services is warranted to reduce the impact of high living costs and financial hardship on mental health.

## Electronic Supplementary Material

Below is the link to the electronic supplementary material.

Supplementary Material 1

## Data Availability

The raw interview transcripts cannot be shared as ethics approval does not allow for this as these include potentially personally identifiable information such as specific locations. The Nvivo file used for the analysis can be shared upon reasonable request.

## Footnotes

1 Silvercloud health is suite of online self-guided Cognitive Behavioural Therapy (CBT) programmes for a range of conditions, such as depression and anxiety. They are used in many NHS talking therapies services (Silvercloud.Amwell.com). The specific Silvercloud programme being referred to here is ‘Space from Money Worries’ (Richardson et al., 2022).

## Declarations

### Patient and Public Involvement

Patient and public representatives helped with the design of these interview guide.
