# Supplementary material for: How do Mental Health Service Users and Staff Think the Cost-of-living Crisis Has Impacted Mental Health in England? A Qualitative Analysis
Source: Community Ment Health J. 2026 Apr 25;62(7):1373–86. doi: 10.1007/s10597-026-01625-6 (PMC13586036; doi:10.1007/s10597-026-01625-6)
Supplement: Supplementary file 1 [file 10597_2026_1625_MOESM1_ESM.docx]

**Supplementary Material**

**Interview/focus group questions for service users:**

Demographics

1. What is your age?
2. What is your gender?
3. How would you describe your ethnicity
4. Are you currently using mental health services? If no, when was the last time?
5. Please share your diagnosis if you have one, and if you are happy to share this information with us.

Financial difficulties during the Cost of Living (CoL) crisis:

1. Did you face any problems with money or paying for things you need because of the increased cost of living? If so, can you tell me about those difficulties?
2. Did you have trouble affording important things like housing, food, or clothes because of the increased cost of living? If yes, could you share what that was like for you?

The impact of the CoL crisis/financial difficulties on MH:

3. Has the current high cost of living affected your mental health and well-being? If so, how?
4. In what ways have money worries or not having enough money affected how you feel and your mental health?
5. Has your mental health impacted your ability or manage your money during the cost-of-living crisis?
6. Have you ever had to make hard decisions or give up things because you didn't have enough money? How did those choices make you feel and affect your mental well-being?

Support seeking:

7. Has your money situation during the cost-of-living crisis affected your ability to get the help and support you need for your mental health? If so, how?
8. Can you describe how you have sought professional help for your mental health during the cost-of-living crisis? Did you get the help you were looking for?
9. Have you received support with your finances during the cost-of-living crisis? If yes, where from? Did this help your mental health?
10. Can you describe the biggest problems/challenges/difficulties you may have faced when seeking professional support regarding your mental health and/or money problems? Did this help?
11. What would help you with both money problems and mental health problems during the current cost of living crisis?
12. What support do you think would benefit those who are struggling with both money problems and mental health problems?

**Interview/focus group questions for staff (NHS / third sector / other):**

Demographics

1. What is your age?
2. What is your gender?
3. How would you describe your ethnicity?
4. Where do you work?
5. What is your job role?
6. How long have you worked in this role?

The impact of the CoL crisis/financial difficulties on MH of service users:

1. Has the cost-of-living crisis impacted the mental health of your services users? Can you describe any examples of this among your service users?
2. In your experience, how has the rising cost of living contributed to mental health challenges for service users? Are there any common patterns you've noticed?
3. Do you think mental health problems impact the finances of your service users? If yes, how?
4. From your perspective, how might financial difficulties and the increased cost of living make existing mental health issues worse?

Service demands and support seeking during the CoL crisis:

5. Have you noticed any major changes in the number of people seeking help from your organisation during the cost-of-living crisis? If so, can you describe these changes?
6. How has the current cost of living crisis affected the types of services/help people ask for from your organisation? Are there any new trends or shifts in what people need?
7. From your experience, how has the increased financial strain on individuals and families affected how often they need your services or how much help they require? Are there any specific areas where demand has gone up or down?
8. In what ways have you seen the cost-of-living crisis affect the kinds of people who come to you for help? Are there any new groups or types of individuals emerging as a result?
9. How do you think the cost-of-living crisis has affected how easily people can access and get the services they need? Have you seen any obstacles or difficulties in meeting the demand for services during this time?
10. How have you supported those with financial difficulties during the cost-of-living crisis?

Improving services:

11. In your opinion, what kind of help or resources do people facing financial difficulties and the high cost of living need to support their mental well-being? Have you seen any effective strategies or interventions that address these challenges?
12. What would make things easier for people to seek and receive the support they need during the cost-of-living crisis?
13. What would have made things easier for you to provide support/do your job during the cost-of-living crisis?
